# Hospital-Acquired Pneumonia and Ventilator-Associated Pneumonia: A Literature Review

**DOI:** 10.3390/microorganisms12010213

**Published:** 2024-01-20

**Authors:** Mihnea Miron, Mihaela Blaj, Anca Irina Ristescu, Gabriel Iosep, Andrei-Nicolae Avădanei, Diana-Gabriela Iosep, Radu Crișan-Dabija, Alexandra Ciocan, Mihaela Perțea, Carmen Doina Manciuc, Ștefana Luca, Cristina Grigorescu, Mihaela Cătălina Luca

**Affiliations:** 1 Faculty of Medicine, University of Medicine and Pharmacy “Grigore T. Popa” of Iași, 700115 Iasi, Romania; miblaj@yahoo.com (M.B.); anca.ristescu@umfiasi.ro (A.I.R.); andrei.n.avadanei@gmail.com (A.-N.A.); diana.iosep@yahoo.com (D.-G.I.); crisanradu@gmail.com (R.C.-D.); mihaela.pertea@umfiasi.ro (M.P.); dmanciuc@yahoo.com (C.D.M.); stefana.luca@d.umfiasi.ro (Ș.L.); cristina.grigorescu@umfiasi.ro (C.G.); catalina_luca2006@yahoo.com (M.C.L.); 2 Anesthesiology and Intensive Care Unit, “Sf. Spiridon” Hospital, 700111 Iasi, Romania; 3 Anesthesiology and Intensive Care Unit, Regional Institute of Oncology, 700483 Iasi, Romania; 4 Anesthesiology and Intensive Care Unit, Clinical Hospital of Pneumology, 700182 Iasi, Romania; gabitito2000@yahoo.com; 5 Pulmonology Department, Clinical Hospital of Pneumology, 700182 Iasi, Romania; 6 Clinical Hospital of Pneumology, 700182 Iasi, Romania; morarualexandra89@yahoo.com; 7 Department of Surgery 1, “Sf. Spiridon” Hospital, 700111 Iasi, Romania; 8 Clinic of Infectious Diseases, “Sf. Parascheva” Clinical Hospital of Infectious Diseases, 700116 Iasi, Romania; 9 Thoracic Surgery Department, Clinical Hospital of Pneumology, 700182 Iasi, Romania

**Keywords:** hospital-acquired pneumonia, ventilator-associated pneumonia, bundle of care, mid-regional pro-adrenomedullin, mid-regional pro-atrial natriuretic peptide, procalcitonin

## Abstract

Hospital-acquired pneumonia (HAP) and its subtype, ventilator-associated pneumonia (VAP), remain two significant causes of morbidity and mortality worldwide, despite the better understanding of pathophysiological mechanisms, etiology, risk factors, preventive methods (bundle of care principles) and supportive care. Prior detection of the risk factors combined with a clear clinical judgement based on clinical scores and dosage of different inflammatory biomarkers (procalcitonin, soluble triggering receptor expressed on myelloid cells type 1, C-reactive protein, mid-regional pro-adrenomedullin, mid-regional pro-atrial natriuretic peptide) represent the cornerstones of a well-established management plan by improving patient’s outcome. This review article provides an overview of the newly approved terminology considering nosocomial pneumonia, as well as the risk factors, biomarkers, diagnostic methods and new treatment options that can guide the management of this spectrum of infections.

## 1. Introduction

Classically, nosocomial pneumonia represents a spectrum of medical conditions that can be classified in two main groups: hospital-acquired pneumonia (HAP) and ventilator-associated pneumonia (VAP), with each having different pathogenesis, risk factors, diagnostic tools and treatment options. While HAP is defined as the infection of the pulmonary parenchyma which appears after at least 48 h of hospital admission, VAP occurs in patients admitted in an intensive care unit (ICU) who required tracheal intubation and mechanical ventilation for at least 48 h [1,2].

**Figure 1:**
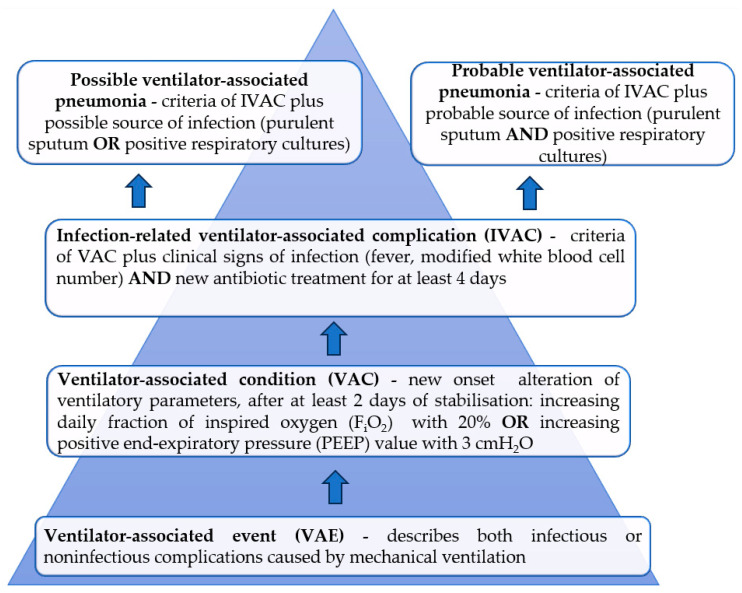
Algorithm of detecting possible/probable ventilator-associated pneumonia with the terminology proposed by the 2013 Centers of Disease Control and Prevention; adapted from [4].

Moreover, taking into consideration the rapidly evolving knowledge about complications of mechanical ventilation, in 2013, the Centers of Disease Control and Prevention of the United States introduced some new medical terms on this topic, with focus not only on the respiratory tract infections, but also including the fraction of inspired oxygen (FiO_2_) and the positive end-expiratory pressure (PEEP) values as surrogates of hypoxemia [3]. Thus, the following terms have been defined, with the main characteristics for each of them being noted in Figure 1: ventilator-associated events (VAE), ventilator-associated condition (VAC), infection-related ventilator-associated complication (IVAC), possible ventilator-associated pneumonia and probable ventilator-associated pneumonia.

Moreover, several new concepts regarding the spectrum of nosocomial pneumonia that occurs in patients admitted in Intensive Care Units (ICUs) have been described, all of them representing different types of ICU—acquired pneumonia. Thus, the infection of the lower respiratory tract which occurs in patients admitted in ICUs for at least 48 h is called ICU-acquired pneumonia, which includes not only VAP, but also HAP in patients that do not require mechanical ventilation. It is also important to understand that, according to their definitions, neither of the subtypes of ICU-acquired pneumonia is present at time of hospital admission or at the onset of tracheal intubation and mechanical ventilation. Recently, authors highlighted the importance of this classification of nosocomial pneumonia in ICU patients, with it being noted that the outcome significantly depends on the type of pneumonia which has been diagnosed. Therefore, one of the key points to be mentioned is that the highest mortality in ICU patients with nosocomial pneumonia is determined by HAP, which subsequently requires tracheal intubation and mechanical ventilation (ventilated ICU-acquired HAP), followed by VAP, while the lowest mortality rate is observed in patients with non-ventilated ICU-acquired HAP [5,6].

**Figure 2:**
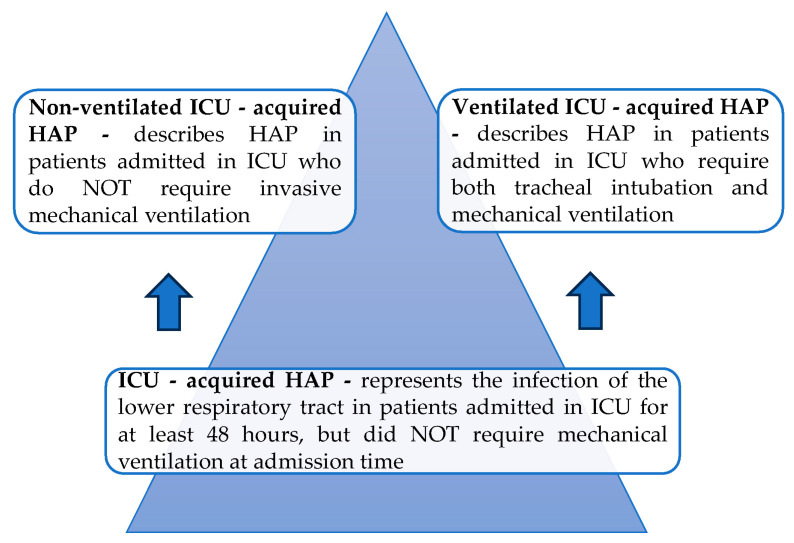
Definitions for ventilated and non-ventilated ICU-acquired HAP; adapted from [5].

Clearly, there are two types of ICU-acquired HAP, depending on the need of mechanical ventilation, and both are defined in Figure 2.

## 2. Materials and Methods

The present article provides a general overview of current knowledge regarding hospital-acquired pneumonia and ventilator-associated pneumonia, comprising the information available in current scientific literature concerning epidemiology, etiology, risk factors, useful biomarkers and diagnostic tools, as well as new treatment options and preventive methods for HAP/VAP management.

The scientific literature was browsed by using the PubMed and UpToDate databases, including the following keywords: “HAP/VAP management”, “VAP terminology”, “HAP/VAP risk factors”, “VAP biomarkers”, “VAP diagnosis”, “HAP/VAP bundle of care” and “HAP/VAP new treatment”. In addition, the terms “HAP” and “VAP” were combined with some other search terms for a particular section of the subject. Our research accessed articles available online that were only written in the English language, including both paid and open access types of articles. Articles with no clear study design or not focusing on HAP and VAP management in adults were also excluded.

## 3. Relevant Sections

We will focus our review on current opinions regarding epidemiology, pathogenesis, risk factors, diagnostic features, novel biomarkers and treatment options used for the management of hospital-acquired pneumonia and ventilator-associated pneumonia, respectively.

### 3.1. Epidemiology

HAP remains the second most common inhospital infection after urinary tract infections (UTIs), with considerable effects on both medical and economical aspects. Moreover, an interesting fact is that even if the incidence number of HAP is greater in non-ventilated patients, the risk of developing this condition is ten times higher in patients requiring mechanical ventilation [7].

On the other hand, because of the inaccuracy of the currently available diagnostic criteria and surveillance methods, the incidence of VAP is not well-established. Although, recent papers cited different incidence rates of VAP depending on the following regions where the studies took place; while in North America the incidence rate is between 1 and 2.5 cases/1000 ventilator-days, European centers reported significantly higher rates, with more than 18 cases/1000 ventilator days, according to an EU VAP/CAP study [8,9].

Another controversial aspect discussed in recent studies is the all-cause mortality rate associated with VAP because of the heterogenous population that was enrolled in different studies. All-cause mortality linked to VAP is considered to be up to 50%, but with an important debate about the degree to which VAP directly affects mortality in intensive care unit (ICU) patients. Moreover, it is generally accepted that VAP correlates with the prolongation of both duration of mechanical ventilation and length of ICU stay [8].

In addition, there are some differences regarding the incidence rate of VAP in various groups of patients, most of the recent studies being focused on traumatized, immunocompromised or oncological patients. Thus, being known that severe trauma in itself represents a major risk factor for VAP, it is important to specify that among all types of traumas, the relationship between traumatic brain injury (TBI) and VAP was specifically studied, as TBI is the leading cause of both death and long-term disability in traumatized patients. Subsequently, in a recent meta-analysis, authors found that the incidence rate of VAP in patients with TBI varies widely between studies, from 21% to 60%, with higher rates of early-onset VAP episodes comparing to the late-onset VAP subtype. Moreover, in the same study there have been described several independent risk factors for VAP occurrence, such as smoking, tracheostomy, blood mass transfusion at hospital admission time, diabetes or high values of injury severity score (ISS) [10]. Another imperative and debatable factor to discuss is that some studies suggest the impact in differentiating VAP in ventilated traumatized patients with trauma-associated pneumonia (TAP), still being unclear if the same diagnostic criteria of VAP used as in non-traumatic patients would significantly change the incidence rate in this population. In addition, some other trauma factors were found in significant correlation with the risk of VAP developing, as multiple rib fractures, pulmonary contusion, sternal fracture and spinal injury [11]. 

Another important category studied is represented by the severely immunosuppressed population, more exactly transplant recipients. Thus, in this group of patients, nosocomial pneumonia is one of the most frequent healthcare-associated infection (HAI), together with bloodstream infections (BSIs), with high mortality and morbidity rates. In patients who received a hematopoietic stem cell transplant, one of the most important risk factors of pneumonia remains neutropenia, the incidence rate varying between 15% to 30%. By contrast, in patients who received a solid organ transplant, the incidence rate depends on the organ which has been transplanted, the highest value (approximately 30%) being observed after pulmonary transplantation [12].

With respect to oncological patients, there is still uncertainty when it comes to epidemiological data regarding healthcare-associated infections, especially pulmonary infections. However, in a recent large retrospective trial that enrolled over 3000 critically ill patients with different types of neoplasia, Stoclin et al. found that increased age, solid tumors, elective surgery and length of mechanical ventilation time are independent risk factors for VAP. Moreover, authors highlighted as a possible limitation of the incidence rate (24.5/ 1000 ventilator days) the severity of patient’s evolution, especially when microbiological diagnosis of VAP was not feasible in patients receiving palliative care [13]. In another study which enrolled postoperative patients diagnosed with head and neck cancer, authors found 4 independent risk factors for VAP: older age, chronic obstructive pulmonary disease (COPD), smoker-status and higher values for Simplified Acute Physiological Score II (SAPS II). Interestingly, in the same study authors have shown that tracheostomy represented a predictive factor for VAP, with an almost threefold decrease in the risk of pulmonary infection [14].

### 3.2. Etiology and Risk Factors

Briefly, HAP and its subtypes can be caused by a wide spectrum of microorganisms and, in some cases, can be polymicrobial, with the most common involved pathogens being methicillin-resistant *Staphylococcus aureus* (MRSA), *Pseudomonas aeruginosa* and other aerobic Gram-negative bacilli, such as *Klebsiella pneumoniae*, *Escherichia coli* and *Acinetobacter baumannii*. Furthermore, it is recognized that in postoperative patients requiring mechanical ventilation, VAP can be produced by viruses, while in immunocompromised patients, both viruses and fungi can cause the infection [8].

Usually clinicians pay more attention to rapidly identify the bacterial cause of the infection since bacteria are frequently isolated in microbiological samples, but authors are encouraging healthcare workers to look after non-bacterial pathogens that are frequently involved in nosocomial respiratory tract infections, like viruses and fungi, as well. Thus, the most frequent incriminated viruses in the etiology of nosocomial pneumonia are the influenza virus and respiratory syncytial virus (RSV), but others are also reported, like adenovirus, rhinovirus, cytomegalovirus, metapneumovirus and parainfluenza virus, with similar or even higher mortality rates compared to bacterial nosocomial pneumonia. Furthermore, there is a continuous debate on Herpes Simplex Virus (HSV) identification from the lower respiratory tract samples in critically ill patients admitted to the ICU, being uncertain if patients are either clearly infected or if the virus was reactivated because of the altered immune system, or even if it was just a contamination from the upper airways [15,16].

Fungal respiratory infection is another concern that increases both morbidity and mortality, especially in immunocompromised hosts, with *Aspergillus* being the most frequently cited in recent studies. It is important to note that the main risk factor for aspergillosis pneumonia is represented by sustained neutropenia, since pulmonary tissue’s granulocytes are the first line cells involved in immune response [17].

Furthermore, in addition to the general aspects regarding the etiology of nosocomial pneumonia discussed above, we consider recent data that is useful to present regarding respiratory tract infections in patients diagnosed with different forms of acute respiratory distress syndrome caused by severe acute respiratory syndrome coronavirus 2 (SARS-CoV-2). Therefore, in a large study including over 700 critically ill patients with SARS-CoV-2 infection, Grasselli et al. reported that VAP had the highest incidence among healthcare-associated infections (HAIs), being followed by bloodstream infections (BSIs) and catheter-related BSIs. Furthermore, the most frequent pathogens which determined VAP were Gram-negative bacteria from the *Enterobacterales* family, together with *S. aureus* as the most frequent Gram-positive pathogen [13]. In another interesting study regarding multidrug resistant VAP before and during the coronavirus disease-19 (COVID-19) pandemic, authors described an increased incidence of MDR VAP during the pandemic, with the following aspects: increased rate of infections with *K. pneumoniae*, higher carbapenem-resistant bacterial pathogens, as well as an increased rate of MRSA compared to the pre-pandemic period [14]. In addition, there are several risk factors that have been associated with VAP in SARS-CoV-2 infected patients, like the need for prone position, less preventive methods used to avoid healthcare-workers with direct exposure, increased duration of invasive mechanical ventilation that is necessary to support the respiratory function in infected patients, immune system dysfunction determined by COVID-19 itself, increased sedation time or higher superinfected pulmonary infarction risk observed in these group of patients [18].

Another important fact raised by recent literature is that pathogens causing VAP can vary depending on some other cumulative risk factors, such as length of stay in the ICU, length of hospitalization, previous exposure to antibiotic treatment, local microbial ecology and timing of ventilatory support [19,20]. Precisely, it is generally accepted that early-onset ventilator-associated pneumonia, which occurs within the first 4 days of ventilatory support, is usually caused by non-multidrug resistant (non-MDR) pathogens, which can be found in the common oropharyngeal flora; in comparison, in the late-onset type, which occurs after at least 5 days of ventilatory support, pathogens are commonly multidrug resistant (MDR) [8,21].

We summarized the main risk factors for HAP/VAP cited in the literature until this moment in Table 1, while in Table 2 we concisely presented the most frequently reported risk factors for MDR pathogens.

### 3.3. Pathophysiology

Several mechanisms which are supposed to cause infection of the pulmonary parenchyma have been described, but better understanding is needed to prevent the infectious pulmonary complications in a critically ill patient who requires mechanical ventilation.

Thus, the development of HAP or VAP depends not only on the number and virulence of the microorganisms entering the lower respiratory tract, but also on a patient’s response, including cellular, humoral and mechanical defense mechanisms. Furthermore, the presence of an endotracheal tube itself represents a significant risk factor for microaspiration from the common oropharyngeal flora, or even from the gastrointestinal tract [7].

Another mechanism which typically occurs in mechanically ventilated patients is represented by an exogenous pathway which includes contaminated environmental sources, such as active humidification systems or different respiratory devices [7,8,22]. Moreover, constantly changing the ventilator circuits in order to control the infectious source is not generally recommended, with the maximum safe duration for using the same circuit being unknown [22]. Contamination during procedures like tracheal suctioning, fiberoptic bronchoscopy or disconnecting the ventilator circuit for patient transport is also included in the exogenous pathogenic route [23].

More precisely, there are several factors that create a complex interaction in the pathogenesis of VAP depending on both host immunity (with impaired molecular pattern altered phagocytosis, T cells’ dysfunction, monocyte deactivation) and the presence of the endotracheal tube (with biofilm formation and impaired mucocilliary clearance) [24].

### 3.4. Diagnostic Tools

Currently, there is not a generally accepted or documented gold standard diagnostic criterion for HAP/VAP. Over time, different clinical and paraclinical methods have been studied to identify hospital-acquired pneumonia and its subtypes, but none of them fulfilled both the specificity and sensitivity required [24]. In addition, the limited accuracy of the tests performed by clinicians to diagnose VAP has significant implications in both the literature research and, more importantly, in the patient’s outcome [24,25].

Using only clinical diagnosis of VAP may overlook approximately one-third of cases in the ICU, and the latest guidelines on the management of VAP recommend a complex strategy which includes both clinical and paraclinical tests in order to identify VAP [1,2,24,25,26]. Conventionally, diagnosing VAP using clinical findings can be challenging due to the non-specific signs and symptoms found in a critically ill patient, this being the reason why authors generally recommend a complex clinical approach. Therefore, most patients suspected of having VAP usually present with the following clinical features after at least 48 h from endotracheal intubation and mechanical ventilation: new and progressive infiltrates on the chest radiography or computed tomography scan, plus at least two of the following features, those being new onset of fever (more than 38 °C), altered value of white blood cell count (leukocytosis > 12.000 cells/mm^3^ or leukopenia < 4000 cells/mm^3^) and the presence of purulent secretions [19]. Some studies also included other clinical findings, such as hypoxemia, tachypnea, hemoptysis, crackles and altered ventilator parameters which increased inspiratory pressures with low tidal volumes [27].

In order to simplify the clinical diagnosis, Pugin et al. composed a clinical score, named Clinical Pulmonary Infection Score (CPIS), that combines both clinical and paraclinical (radiological and laboratory) parameters [28]. However, according to the latest studies on VAP, neither the sensitivity nor the specificity of the score exceeds 60%, and because of this, integrating CPIS in clinical practice remains a matter of controversy [27]. It was initially considered that a score above six points represents the specific threshold value for detecting VAP, but recent prospective cohort studies failed to demonstrate any significant correlation [27,28].

In Table 3, we present the parameters included in CPIS, with the respective scoring system.

Nonetheless, the latest version of the European guidelines for the management of HAP/VAP, as well as the American Thoracic Society (ATS) and the Infectious Diseases Society of America (IDSA) guidelines, recommend qualitative or quantitative cultures from microbiological samples obtained from the lower respiratory tract as a cornerstone in identifying and diagnosing VAP [2,24,25,26]. Moreover, according to the technique used for obtaining samples, there are two different methods accepted in the literature, those being invasive methods which require fiberbronchoscopy and non-invasive methods which do not require bronchoscopic guidance [24].

In Table 4 we summarized different techniques that can be performed for obtaining microbiological samples from the lower respiratory tract, depending on the invasive or non-invasive method used.

Another important fact to mention is that each of the techniques used to obtain the microbiological samples has a diagnostic threshold value expressed in colony forming units/mL (CFU/mL), such as 103 CFU/mL for PSB, 104 CFU/mL for BAL or 106 CFU/mL for endotracheal aspirates [24,27]. After the moment samples are obtained, they are sent for a proper microbiological exam (gram stain, culture identification and antibiotic sensitivity) and the results are either expressed in qualitative or quantitative values [24]. Another useful piece of information is about whether the sample obtained is purulent or not, which is found by identifying the amount of neutrophils and squamous epithelial cells per low power field (at least 25 neutrophils and less than 10 squamous epithelial cells are typically observed in purulent specimens) [24].

To increase the accuracy of the diagnostic methods, several biomarkers were studied, with none of them being considered the gold standard for HAP/VAP: procalcitonin, C-reactive protein (CRP), soluble triggering receptor expressed on myelloid cells type 1 (sTREM-1) and mid-region fragment of pro-adrenomedullin (MR pro-ADM). Generally, biomarkers represent proteins whose presence should correlate not only with the severity of the disease, but also with patient’s outcome, as a useful diagnostic tool [29]. Moreover, an ideal biomarker for VAP should significantly corelate with the other diagnostic criteria presented above in any case of ventilator-associated pulmonary infection, with high sensitivity and specificity for a proper diagnosis. However, diagnostic prediction of VAP remains uncertain, and more clinical trials are needed to determine the usefulness of any biomarker in clinical practice [30].

Subsequently, we present some general aspects regarding biomarkers studied for VAP detection.

Procalcitonin (PCT) represents a widely used and studied serum biomarker which can differentiate bacterial infection from other etiological causes of infection or inflammation. Moreover, regarding VAP, procalcitonin has both advantages and disadvantages; it can be used to guide the antibiotic therapy, being especially useful to consider discontinuation of antibiotherapy (when the serum levels are less than 0.5 ng/mL or the value has decreased by at least 80% from the peak level according to a ProVAP randomized trial) and also as an adjunct when diagnosis of VAP is uncertain. In addition, it is generally recommended to measure serial PCT values and not a single level during an ICU stay, in order to determine the rising or falling trend of the values [31]. The main disadvantages reported in measuring PCT are the influence of previous antibiotic exposure on serum levels and that there is no optimal threshold value which can guide starting the antibiotherapy, although latest guidelines suggest to start antibiotherapy at the moment of suspicion of VAP, regardless of the level of PCT [29,30,31]. Another limitation is that the extent to which PCT values rise is dependent on the pathogen, with higher values observed in infections caused by typical bacteria [31].

C-reactive protein (CRP) is another biological marker which can detect inflammation, being synthesized by the liver. The normal serum level in healthy people is around 1 mg/L, but the values can immediately raise in the case of several conditions, such as bacterial infection, postoperative status, burns or neoplasia, making CRP a non-specific marker of inflammation. Several trials have compared serum CRP levels with bronchoalveolar lavage CRP levels, but results still remain unclear [29].

A soluble triggering receptor expressed on myelloid cells type 1 (sTREM-1) is a complex biomarker which represents a glycoprotein belonging to the immunoglobulin G (Ig G) superfamily, and its expression on phagocytes is dependent on bacterial or fungal exposure [29]. One of the physiological functions of this biomarker is the augmentation of the pro-inflammatory response by modulating the secretion of several pro-inflammatory mediators, which is the reason why sTREM-1 can be found and measured in body fluids (blood, BAL) [29]. Some studies conducted in the past demonstrated that sTREM-1 values measured from BAL are higher in patients suspected of VAP in comparison to non-VAP patients, but the usage of sTREM-1 as a specific marker for VAP was not conclusive, due to both heterogenous criteria used for defining VAP and different methods applied for obtaining lower respiratory tract samples [29,32].

Mid-regional pro-atrial natriuretic peptide (MR pro-ANP) represents the N-terminal part of the prohormone of atrial natriuretic peptide (ANP), which is secreted in the same quantity as the mature molecule ANP after it has been cleaved [33]. Recently, plasma MR pro-ANP levels were studied in patients with either sepsis-induced myocardial depression or acute lung injury, and some authors demonstrated a possible correlation between plasma levels and the cytokine response in pneumonia, dependent on the severity of the illness [33]. Moreover, it seems that, according to recent clinical trials, MR pro-ANP was the only independent predictor of a 28 day mortality rate, in comparison to some other parameters evaluated, such as an APACHE II score, serum creatinine levels, age or gender [33]. In addition, Boeck et al. demonstrated a statistically significant correlation between plasma MR pro-ANP levels and PCT serum levels regarding improvement of the survival rate in patients diagnosed with VAP [34].

Mid-regional pro-adrenomedullin (MR pro-ADM) is another novel and complex biomarker which has recently gained interest in detecting whether or not it can help the clinician in evaluating not only different forms of infection, but also organ failures and complications in different comorbidities (acute dyspnea, ARDS). Consequently, MR pro-ADM represents a peptide fragment derived from adrenomedullin with proper physiological functions, such as immune modulating, metabolism and vasodilation [35,36]. Furthermore, Spoto et al. demonstrated a significant correlation between serum levels of PCT and MR pro-ADM in risk stratification of patients with pneumonia, while Önal et al. evaluated the impact of MR pro-ADM and its prognostic role in sepsis and septic shock [35,37]. Other recent studies evaluated the prognostic value of the biomarker in patients with ARDS or pneumonia, with MR pro-ADM levels being useful for stratifying mortality risk and evaluating other forms of organ failure that can occur [38,39].

### 3.5. Preventive Methods

The next section will be dedicated to different recommended methods used in clinical practice to prevent HAP/VAP. Thus, some recent studies have noted that preventing HAP and VAP would substantially contribute to reducing both medical complications and economical problems raised by these conditions.

Therefore, the most important preventive methods in reducing HAP and VAP incidence are the following: preventing pulmonary aspiration of oropharyngeal secretions (by raising the head of the bed to 30°–45°, daily sedation interruption, maintaining a constant airway cuff pressure of the endotracheal tube to a maximum of 30 cm of H2O, applying of optimal positive end-expiratory pressure—PEEP); subglottic secretions drainage with specially designed devices which permit either continuous or intermittent aspiration; measurement of gastric residual volume (with no documented impact on aspiration risk); regular oropharyngeal care with chlorhexidine (being statistically correlated with a reduction in HAP incidence in selected cases); selective decontamination of the digestive tract (SDD) with colistin; tobramycin or nystatin; vaccination against *H. influenzae* and *S. pneumoniae* of both patients and healthcare workers (as these pathogens seem to exacerbate a HAP/VAP episode) and proper treatment with proton-pomp inhibitors and histamine2 (H2) receptor blockers (especially in older, postoperative patients) [21,40].

Recently, authors implemented the concept of “bundle of care”, which represents a combination of different techniques, the most important ones being mentioned above, with the main purpose being the reduction of HAP/VAP incidence, alongside the complications that can occur in critically ill patients. Therefore, several studies encourage the implementation of standardized protocols in the ICU regarding preventive methods, with a significant impact on lowering especially the late-onset episodes of VAP [41].

In another revised study, Klompas et al. highlighted some other recommendations that should be included in a standardized “bundle of care” for preventing VAP, with the quality of evidence (as high, moderate or low) being mentioned for each method as well. Therefore, among preventive methods with a high quality of evidence, authors emphasized the following techniques: using non-invasive modes of ventilation whenever suitable (high-flow nasal oxygen or non-invasive positive pressure ventilation) to avoid endotracheal intubation, providing early enteral nutrition to avoid malnutrition and replacing the ventilator circuit only in selected cases [42].

In a recent meta-analysis, which included 36 significant studies, Reviejo et al. found a correlation between the application of care bundles in order to reduce VAP episodes and duration of invasive mechanical ventilation, but with no statistical relevance concerning the length of hospitalization or the hospital mortality rate [43].

### 3.6. Treatment

Lately, the main goal in managing not only nosocomial pneumonia, but also the spectrum of healthcare-associated infections (HAIs) is to prevent, or even to avoid, antimicrobial resistance mechanisms, with respect to an antibiotic stewardship concept. Recent studies reported that more than 50% of antibiotic drug prescriptions in ICUs are for ventilator-associated pneumonia, highly contributing to the emergence of antimicrobial resistant pathogens worldwide. Additionally, the most important mechanisms of antimicrobial resistance observed in ICU-admitted patients diagnosed with VAP are the change of antimicrobial target, altered permeability or efflux pumps which can reduce the intracellular drug concentration and enzymatic drug inactivation, all of them being responsible for the occurrence of multidrug resistant (MDR) or even extremely drug resistant (XDR) pathogens causing VAP [44].

Traditionally, the key to success in HAP/VAP management is represented by intravenous antibiotherapy, with two main steps which should be followed: the first one is the empirical treatment which respects both the severity of the disease and the presence of risk factors for MDR pathogens (listed in Table 2), while the second step is represented by target or definitive treatment, according to microbiological susceptibility reports or antibiograms [8].

In 2017, Torres et al. included in the latest version of the European guideline regarding the management of HAP/VAP an algorithm concerning the empirical antibiotic treatment for patients diagnosed with HAP or VAP, based on both the presence of risk factors for MDR pathogens and mortality risk. Thus, for patients with no MDR risk factors and low mortality risk (≤15%), authors recommend monotherapy with narrow-spectrum drugs against both methicillin-sensitive *S. aureus* (MSSA) and non-resistant Gram-negative bacilli, by using one of the following agents: ertapenem, ceftriaxone, cefotaxime, levofloxacin or moxifloxacin. On the other hand, for patients at high risk for MDR infections and a >15% mortality risk, the empirical treatment is guided by the presence or absence of septic shock. In the absence of septic shock, a single agent against Gram-negative bacteria (especially *P. aeruginosa*) can be used by choosing between imipenem, meropenem, ceftazidime, cefepime, levofloxacin or piperacillin–tazobactam. If patients are treated in ICUs with high rates (>25%) of methicillin-resistant *S. aureus* (MRSA) in respiratory isolates, vancomycin or linezolid should be added to the initial choice of antibiotic treatment. Furthermore, high risk patients with septic shock should receive a combination between two agents against *P. aeruginosa* (one antipseudomonal betalcactam–imipenem, meropenem, cefepime, ceftazidime, piperacillin–tazobactam or aztreonam, plus either one aminoglycoside–amikacin, gentamicin, tobramycin or one antipseudomonal quinolone–ciprofloxacin, levofloxacin) and one agent against MRSA [1,2].

Concerning pathogen-specific or definitive treatment, the main goal that should be approached in critically ill patients is to avoid the overuse of antibiotics by taking into consideration the following rules: stopping antibiotics when no microorganisms are documented, changing the antibiotic treatment according to available culture results and susceptibility tests, stopping anti-MRSA agents if MRSA is not present, using carbapenems only for susceptible pathogens (carbapenem-only susceptible *P. aeruginosa* or *Acinetobacter* spp.) and proper duration of antimicrobial treatment based on procalcitonin kinetics (antibiotherapy should not exceed 7 days in the majority of cases) [3]. Moreover, recent studies highlighted the significance of the newly available microbiological tests (multiplex polymerase chain reaction—MPCR, chromogenic tests, analysis of the exhalome) in order to reduce antibiotherapy duration and also to properly prescribe antibiotics to critically ill patients by detecting both drug resistance genes and enzymes [45,46].

Furthermore, due to the increased prevalence of antibiotic-resistant pathogens in ICUs (in particular, *Enterobacterales* and non-fermenting Gram-negative bacteria like *P. aeruginosa*, *A. baumannii*), several clinical trials have recently emphasized new antibiotic options for HAP/VAP treatment. Therefore, some general aspects of the newly approved antibiotics for HAP/VAP are presented below.

Ceftobiprole represents a new fifth-generation cephalosporin for HAP, but excludes VAP. The antibacterial spectrum includes MRSA, *Haemophilus influenzae*, *Moraxella catarrhalis*, penicillin-resistant *Streptococcus pneumoniae* (PRP), non-extended spectrum β-lactamase (non-ESBL) and non-carbapenemase producing *Enterobacterales*. One disadvantage of this drug is the fact that it is degraded by both extended spectrum β-lactamase (ESBL) and carbapenemase, being inactive in treating infections caused by anaerobic Gram-negative bacteria, *Enterococcus faecium*, *Acinetobacter baumannii*, *Stenotrophomonas maltophilia* or *Proteus vulgaris* [47].

Ceftolozane–tazobactam represents a combination between an antipseudomonal fifth-generation cephalosporin with a well-defined β-lactamase inhibitor (BLI). The main advantage of this combination is that the antibiotic can reach its target protein (penicillin-binding protein—PBP) after the inhibition of beta-lactamases induced by tazobactam. Recent several trials showed significant results in patients infected with MDR or even extremely drug resistant (XDR) strains of *P. aeruginosa*, ESBL-producing *Enterobacterales*, but with limited activity against anaerobic pathogens, Gram-positive cocci and *Acinetobacter baumannii* [47,48].

Meropenem–vaborbactam is another novel combination of BLI with a carbapenem, approved for both HAP and VAP treatment. It is recognized that vaborbactam is an efficient inhibitor of Ambler classes A and C of β-lactamases, but with no activity against classes B and D; the authors recommend this combination for treating respiratory tract infections caused by carbapenem-resistant *Enterobacterales* (CRE). However, a disadvantage observed in clinical trials is that vaborbactam cannot enlarge the spectrum of meropenem for *A. baumannii* and *P. aeruginosa* [47,48,49].

Ceftazidime–avibactam is a combination of a new non-β-lactam BLI and a third-generation cephalosporin which was approved for HAP/VAP treatment in 2018. As an advantage beyond other BLIs, avibactam has certified activity against Ambler classes A, C and some of the class D enzymes, being useful in treating respiratory infections caused by MDR *P. aeruginosa* strains and ESBL-producing *Enterobacterales*. However, avibactam does not present any activity against Ambler class B β-lactamases, commonly named metallo-β-lactamases [48,50].

Imipenem–relebactam represents a recently approved combination for HAP/VAP treatment between a novel BLI, which is structurally linked to avibactam, and a carbapenem, with potent activity on Ambler classes A and C, but with limited efficacy against Ambler class D and no documented activity on metallo-β-lactamases. However, the spectrum of this combination includes both ESBL-producing *Enterobacterales* and *P. aeruginosa*, but no activity against *A. baumannii* or *S. maltophilia* has been certified yet [47,48,51]. For this reason, the main recommendation for using the imipenem–relebactam combination remains respiratory infections caused by carbapenem-resistant Gram-negative bacilli [47].

Cefiderocol is a new siderophore cephalosporin with innovative characteristics, being approved for HAP/VAP treatment in 2020. The main advantages of this drug include its stability against all of the Ambler classes of β-lactamases, including metallo-β-lactamases, favorable tolerability, a safety profile with minimal side effects, being recommended in infections caused by carbapenem-resistant and MDR Gram-negative bacteria, including *A. baumannii* or *S. maltophilia* [47,48,52,53,54]. However, an important fact to mention is the higher mortality rate observed in critically ill patients with MDR Gram-negative bacterial infections treated with cefiderocol compared to other antibiotic agents, and more clinical trials are required to clarify the exact cause of this aspect [48,53,55]. Moreover, authors cited some particular side effects after using cefiderocol, such as hypokalemia, hypomagnesemia, atrial fibrillation, increased liver enzymes and candidiasis [48].

Moreover, there are some other new antibiotic agents under evaluation concerning HAP/VAP treatment (ceftaroline–avibactam, plazomicin, aztreonam–avibactam, eravacycline, iclaprim or murepavadin) that are expected to have the final results from different phase 3 clinical trials, in order to be approved in the near future [47].

## 4. Future Directions

Because of the significant impact of nosocomial pneumonia and its subtypes on both morbidity and mortality in critically ill patients, more prospective, randomized trials are encouraged in order to detect new risk factors and biomarkers (with proper threshold values) that correlate not only with the clinical evolution, but also with the patients’ outcome. Furthermore, the new microbiological tools which can early detect viral and fungal respiratory tract infections, especially in immunocompromised patients, should be integrated in diagnostic algorithms in order to limit antibiotic resistance mechanisms. Moreover, prospective trials regarding new treatment options, such as microorganism-specific antibodies which can neutralize different virulence factors, are also awaited.

## 5. Conclusions

Despite the ongoing rapid progress which has been made regarding both management and diagnostic methods of HAP/VAP, nosocomial pneumonia and its spectrum remains one of the most significant hospital-acquired infections, resulting in increased mortality, especially in critically ill patients.

A complex and standardized approach is recommended for diagnosing HAP/VAP, including not only the clinical scores, but also the microbiological tools and the kinetics of serum biomarkers, in order to properly identify and evaluate patients with lower respiratory tract nosocomial infections.

The application of “bundle of care” concepts is recommended to prevent HAP and VAP episodes, with better clinical outcomes in patients at risk for lower respiratory tract infections.

The most significant goals in HAP/VAP management are the early administration and appropriate duration of empirical antibiotherapy, followed by de-escalation once the microbiological culture results are available, in order to reduce both antibiotic overuse and the emerging phenomenon of antibiotic resistance. The maximum recommended duration of antibiotherapy is 7 days in the majority of cases.

## Figures and Tables

**Table 1:** Risk factors for HAP/VAP; adapted from [21].

Risk Factors for Developing HAP/VAP
Invasive mechanical ventilation
Extreme age
Altered conscious level
Chronic lung disease, chronic kidney disease
Severe trauma
Malnutrition
Previous exposure to wide-spectrum antibiotics
Aspiration
Prolonged surgical procedures (thoracic/upper abdominal)
Use of glucocorticoids/opioids/neuromuscular blocking agents
Stress ulcer prophylaxis
Acute respiratory distress syndrome (ARDS)
Anemia

**Table 2:** Risk factors for MDR pathogens; adapted from [16].

Risk Factors for MDR Pathogens
ARDS before VAP
Intravenous wide-spectrum antibiotic use in the last 3 months
Septic shock at the moment of suspecting VAP
At least 5 days length of hospitalization before suspecting VAP (late-onset subtype)
Renal replacement therapy before VAP

**Table 3:** Clinical Pulmonary Infection Score (CPIS); modified after [28].

Parameter/Value	0 Points	1 Point	2 Points
Tracheal secretions *	Few	Moderate	Large
Chest radiography infiltrates	No new infiltrates	Diffuse new infiltrates	Localized new infiltrates
Temperature (°C)	36.5–38.4	38.5–38.9	>38.9 or <36
Hypoxemic index (P_a_O_2_/F_i_O_2_ mmHg)	>240 or ARDS		<240 and NO ARDS
White blood cell count (×10^3^/µL)	4–11	<4 or >11	
Microbiological culture	Negative		Positive

* if tracheal secretions are purulent, clinicians should add one more point to the final score.

**Table 4:** Methods for obtaining lower respiratory tract samples.

Invasive techniques	Bronchoalveolar lavage (BAL)
	Mini-bronchoalveolar lavage (mini-BAL)
	Protected specimen brush (PSB)
Non-invasive technique	Endotracheal aspirate

## Data Availability

Not applicable.

## Abbreviations

HAP	hospital-acquired pneumonia;
VAP	ventilator-associated pneumonia;
FiO_2_	fraction of inspired oxygen;
PEEP	positive end-expiratory pressure;
VAE	ventilator-associated events;
VAC	ventilator-associated conditions;
IVAC	infection-related ventilator-associated complications;
UTIs	urinary tract infections;
ICU	intensive care unit;
MRSA	methicillin-resistant *Staphylococcus aureus*;
RSV	respiratory syncytial virus;
HSV	Herpes Simplex virus;
SARS-CoV-2	severe acute respiratory syndrome coronavirus 2;
COVID-19	coronavirus disease-19;
HAIs	healthcare-associated infections;
BSIs	bloodstream infections;
MDR	multidrug resistant;
MDR VAP	multidrug resistant ventilator-associated pneumonia;
ARDS	acute respiratory distress syndrome;
CPIS	clinical pulmonary infection score;
ATS	American Thoracic Society;
IDSA	Infectious Diseases Society of America;
BAL	bronchoalveolar lavage;
PSB	protected specimen brush;
CFUs	colony forming units;
CRP	C-reactive protein;
sTREM-1	soluble triggering receptor expressed on myelloid cells type 1;
MR pro-ANP	mid-regional pro-atrial natriuretic peptide;
ANP	atrial natriuretic peptide;
MR pro-ADM	mid-region fragment of pro-adrenomedullin;
PCT	procalcitonin;
Ig G	immunoglobulin G;
SDD	selective decontamination of digestive tract;
MPCR	multiplex polymerase chain reaction;
PRP	penicillin-resistant *Streptococcus pneumoniae*;
non-ESBL	non-extended spectrum β-lactamase;
ESBL	extended spectrum β-lactamase;
BLI	β-lactamase inhibitor;
PBP	penicillin-binding protein;
XDR	extremely drug resistant;
CRE	carbapenem-resistant *Enterobacterales*.
